# Utilizing conductivity of seawater for bioelectric measurement of fish

**DOI:** 10.1038/s41598-020-73485-3

**Published:** 2020-10-01

**Authors:** Tsunemasa Saiki, Yukako Takizawa, Kazutaka Miyahara, Masakazu Arima

**Affiliations:** 1 Materials and Analysis Department, Hyogo Prefectural Institute of Technology, 3-1-12, Yukihira, Suma, Kobe, 654-0037 Japan; 2 Graduate School of Engineering, University of Hyogo, 2167, Shosha, Himeji, 671-2201 Japan; 3 Manufacturing Technology Department, Hyogo Prefectural Institute of Technology, 3-1-12, Yukihira, Suma, Kobe, 654-0037 Japan; 4 Fisheries Technology Institute, Hyogo Prefectural Technology Center for Agriculture, Forestry and Fisheries, 22-2, Minamifutami, Futami, Akashi, 674-0093 Japan; 5 Graduate School of Engineering, Osaka Prefecture University, 1-1, Gakuen-cho, Naka-ku, Sakai, 599-8531 Japan

**Keywords:** Biological techniques, Ecology, Zoology, Ocean sciences, Health care, Engineering

## Abstract

To manage health conditions of farmed fish and other living creatures, a simple method to measure bioelectric signals of the creatures in seawater is expected. A novel method to measure bioelectric signals by utilizing the conductivity of seawater surrounding the entire body of a fish is proposed. As for the proposed method, a needle-type internal electrode is inserted into the fish’s muscle at a certain measurement point, and an external electrode is sunk in seawater. The internal electrode is isolated from the seawater by virtue of being inserted in the fish. Bioelectric signals generated between the external and internal electrodes are then measured. By sharing the external electrode with the internal electrode, it is possible to measure bioelectric signals with half the number of bioelectrodes used by conventional methods. To demonstrate the practicality of the proposed method, two internal electrodes were inserted into different parts (above the gills and near the tail) of three fish (*Parajulis poecilepterus*, ca. 20 cm fork length) kept in a tank. The proposed method obtained reliable bioelectric signals corresponding to electrocardiograms (ECGs) and electromyograms (EMGs) from each part of the individual fish.

## Introduction

For sustainable use of marine-animal resources, preservation of endangered species, and conservation of ecosystems, it is very important to understand the biology of individual marine animal. From the viewpoints of physiology, ethology, and environmentology, marine animals have been studied by bioelectric measurement^1–4^, bio-logging^5–9^, and DNA (genome) analysis^10–15^, respectively. Recent technological innovations helped studies on bio-logging and DNA analysis advance rapidly, but advancement of bioelectric-measurement technology, which has existed for a long time, lags behind those of bio-logging and DNA analysis.

Now, aiming to obtain good harvests, the aquaculture industry requires bioelectric measurements to grasp the health condition of marine animals from pathophysiological viewpoints. Moreover, the electrocardiogram (ECG), which is a kind of bioelectric measurement, carries high expectations because it can evaluate psychological stress of marine animals just as it can evaluate that of humans^16–19^. Moreover, ECG can be used in fish ethological- and physiological studies^2,4^, so innovating techniques and devices for ECG measurement will contribute to developing these studies.

In regards to bioelectric measurement targeting marine animals, to prevent electric short-circuiting between the pair of bioelectrodes via seawater (which is conductive), one or multiple pairs of bioelectrodes are embedded inside the living body by incision surgery^20,21^, which can impose a heavy workload on inexperienced experimenters. Moreover, the animal can often become agitated without anesthesia and consume much physical energy when the electrodes are implanted into its body. To reduce these burdens, we propose a novel method of measuring bioelectric signals—which utilizes the conductivity of seawater surrounding the animal—by using only one bioelectrode attached at each measurement point (in contrast to the conventional method, which requires a pair of bioelectrodes). To the best of our knowledge, a similar method has not been reported.

In this paper, the proposed method of bioelectric measurement for marine animals under the seawater is first overviewed. Next, the bioelectric measurement system for the chosen experimental subjects, namely, fish, is described, and the availability of the proposed method is verified. Then, the experimental procedures and results of bioelectric measurements are presented. Finally, possible applications of the proposed method are discussed.

## Proposed method of bioelectric measurement

**Figure 1:**
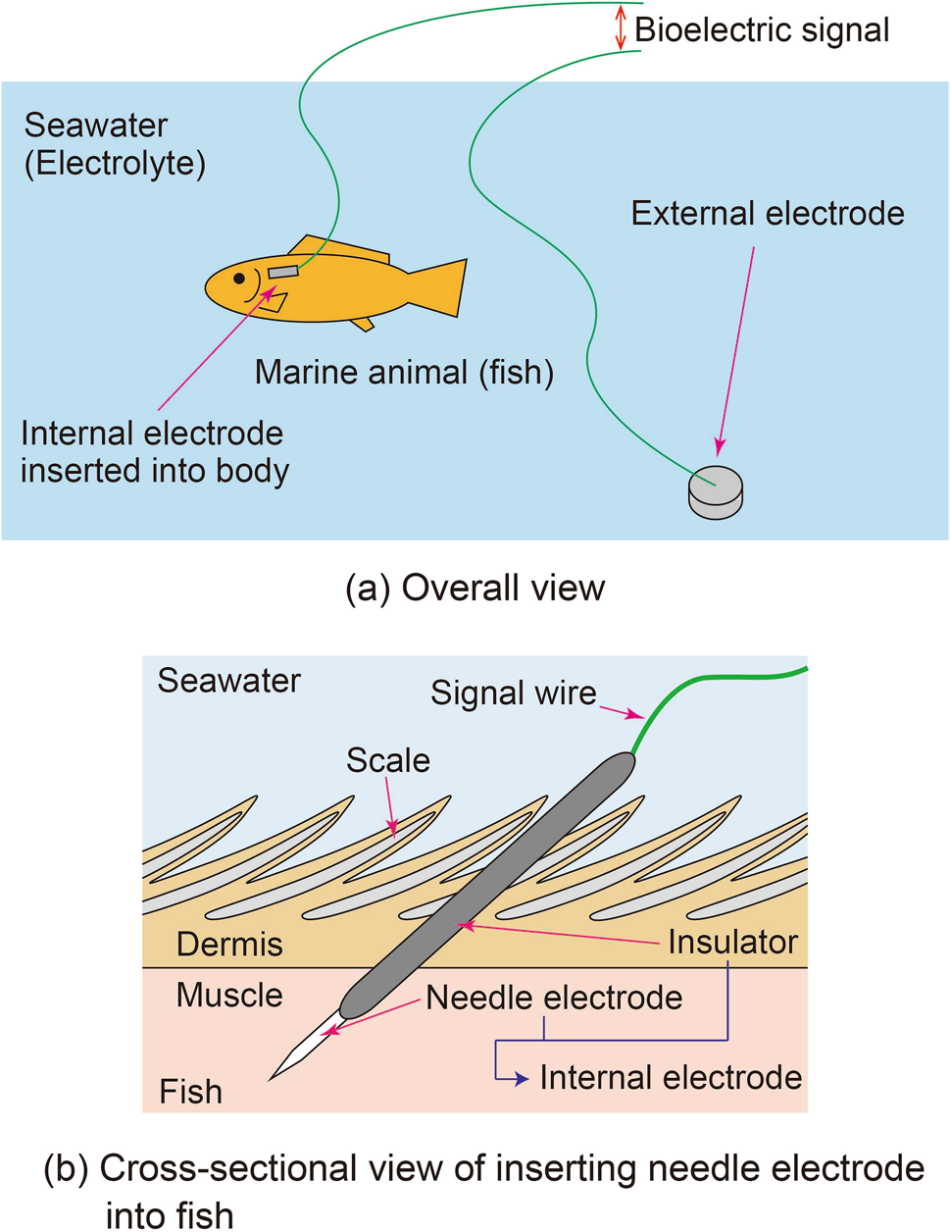
Principle of bioelectric measurement utilizing conductivity of sweater for marine animals.

The proposed method (Fig. 1a) employs seawater as an electrolyte (conductive liquid), in which an external electrode is sunk. An internal electrode is inserted at a measurement point on the marine animal, e.g., a fish, to be measured. The internal electrode is a needle-type electrode insulated on the signal-wire side (Fig. 1b). By inserting the internal electrode from outside of the fish’s body into a muscle through the dermis, the conductive part on the needle tip is isolated from the seawater. In this manner, bioelectric signals generated between the external electrode and the internal electrode can be measured.

**Figure 2:**
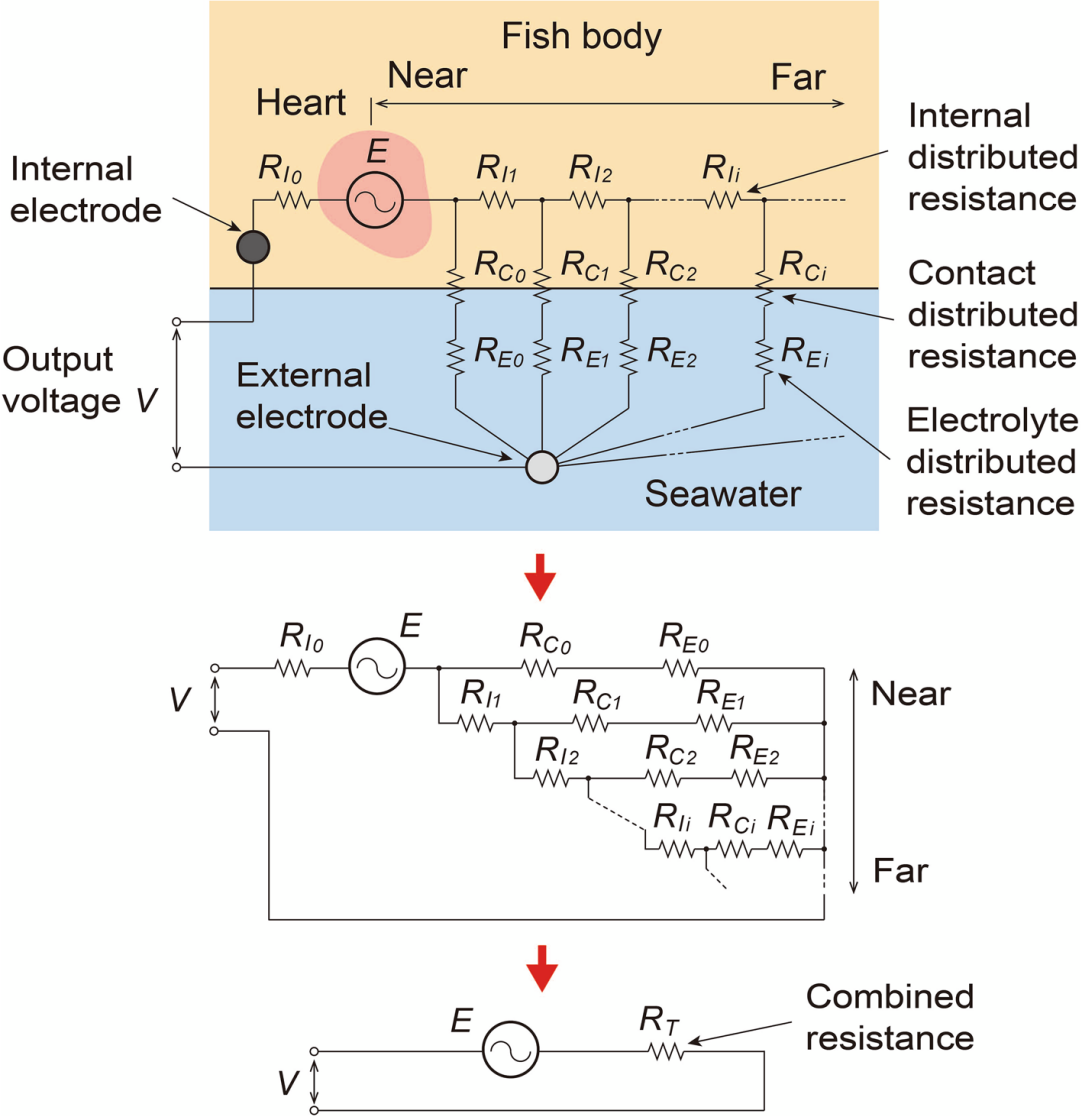
Simplified electric-circuit model of bioelectric-measurement system utilizing seawater.

An electric circuit model (Fig. 2) is used to logically explain the reason that bioelectric signals can be obtained through the external and internal electrodes as follows. Although this model is very simple, the actual measurement system utilizing the proposed method is a complicated three-dimensional distributed circuit, in which, *E* is a cardiac action potential, and *R*_*Ii*_, *R*_*Ci*_, and *R*_*Ei*_ are internal, contact, and electrolyte (seawater) distributed resistances, respectively. Since the electric circuit consists of *E*, *R*_*Ii*_, *R*_*Ci*_, and *R*_*Ei*_, it becomes possible to obtain output voltage *V*, i.e., the bioelectric signal, through the external and internal electrodes.

When the above-described electric-circuit model is used to consider the features of the proposed method, it becomes clear that as the surface area of the fish increases, the number of resistors *R*_*I*_, *R*_*C*_, and *R*_*E*_ (connected in parallel) increases, i.e., the combined resistance *R*_*T*_ decreases; thus, the obtained bioelectric-signal voltage increases. However, it should be noted that *R*_*Ii*_, *R*_*Ci*_ and *R*_*Ei*_ with larger *i*, i.e., ones which are farther from the heart, have smaller effects on reducing *R*_*T*_. On the contrary, when the distance between the fish and the external electrode becomes shorter, all values of *R*_*E*_ become smaller; thus, the value of *R*_*T*_ becomes smaller, i.e., the bioelectric-signal voltage becomes larger.

In the case of multipoint bioelectric measurement by the proposed method, the number of necessary bioelectrodes embedded in fish’s body can be reduced by a half compared with the conventional method (which requires a pair of bioelectrodes at one measurement point). Since the external electrode can be used as a common bioelectrode, only one bioelectrode is attached at each measurement point. Each bioelectrode can be inserted from outside the fish’s body, while conventional methods occasionally require open surgery to install the bioelectrode in the inner body. In other words, the proposed method enables very simple bioelectric measurement that can reduce the workload on experimenters and the physical stress of marine animals.

## Measurement system

**Figure 3:**
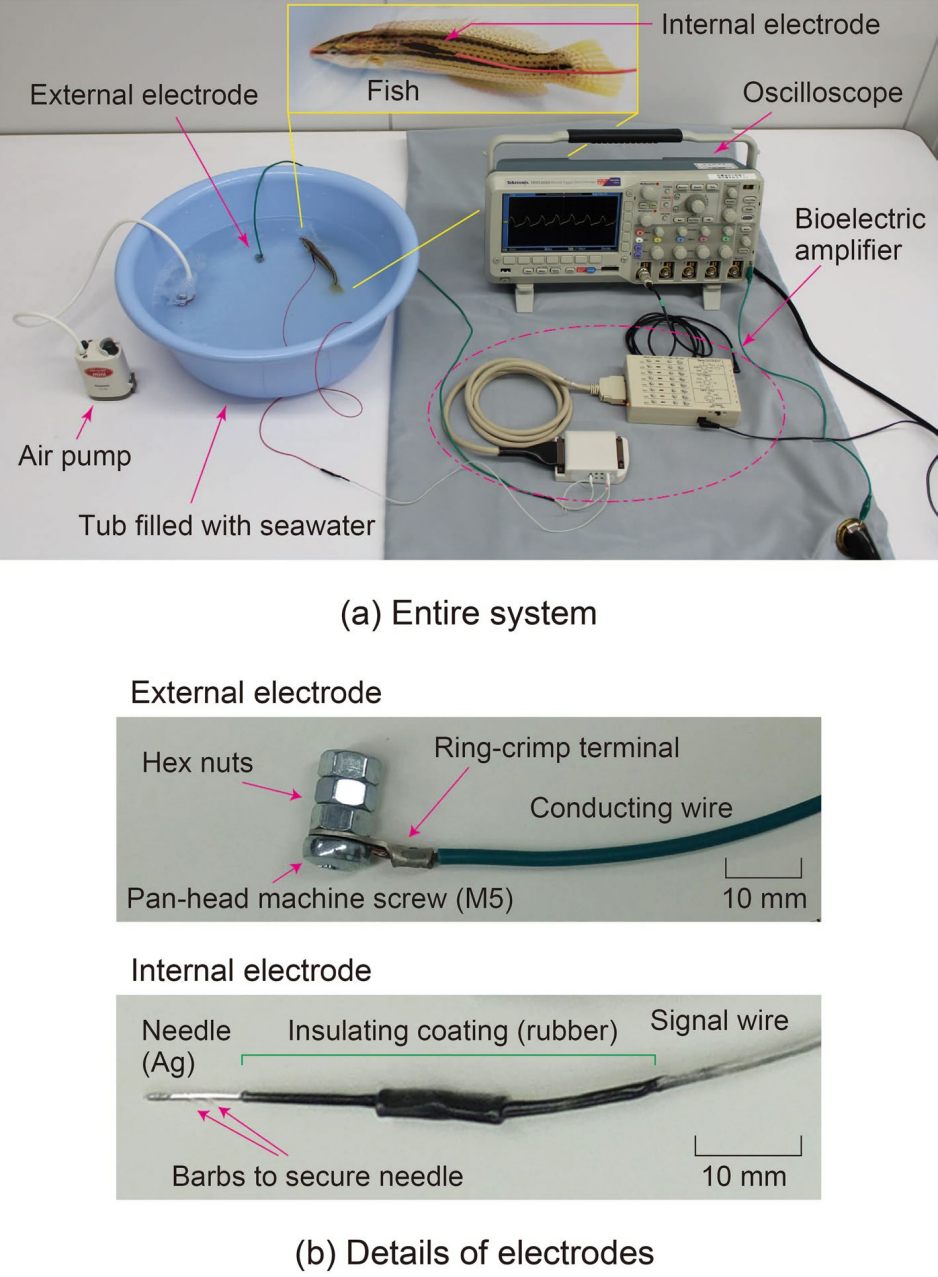
(**a**) Photograph of the entire experimental system for verifying the bioelectric measurement utilizing conductivity of seawater and (**b**) details of the bioelectrodes used in the experiment.

The experimental system used to verify the proposed method—which utilizes the conductivity of seawater for bioelectric measurement of an aquatic animal (i.e., a fish)—is shown in Fig. 3a. The system consists of four parts: (1) a live fish in a plastic tub (about 36-cm opening diameter), (2) special bioelectrodes (external and internal electrodes) for detecting electrical signals of the fish generated in vivo, (3) a bioelectric amplifier for amplifying the detected electrical signals, and (4) equipment for observing and recording the amplified signals.

The tub was filled with six liters of natural seawater (water temperature: 20 °C; electrical conductivity: 5.0 S/m) sampled from Osaka Bay where the subjects (fish) were captured. The seawater was continuously supplied with air by pump during the experiment. The external electrode (Fig. 3b) consisted of a ring-crimp terminal (tin-plated copper), a pan-head machine screw (size M5, unichrome-plated iron) and three hex nuts (size M5, unichrome-plating iron). The external electrode also worked as a sinker and was stably placed on the bottom of the tub. The internal electrode (Fig. 3b) was made by connecting a signal wire (AWG24) with a silver conducting wire (0.8-mm wire diameter) with a sharply pointed tip for inserting into the fish’s body and two barbs to secure the tip by a crimp sleeve (Nichifu Co., B-0.5). Except for the 10-mm-long tip, the electrode was coated with insulative rubber paint (PDI Inc., PROT). Incidentally, the resistance between the internal and external electrodes left in the seawater did not change over several hours. The resistance between the bioelectrodes was measured by a resistance meter specialized for bioelectric measurement (Nihonsanteku Co., MaP811). This result confirmed that almost no electrochemical reaction occurs in seawater; it is thus considered that the measurement system using the bioelectrodes is stable.

Bioelectric signals detected from the external and internal electrodes were amplified a thousand times and processed with high-pass and low-pass filters by a bioelectric amplifier (TEAC Co., BA1008). The amplified and processed bioelectric signals were then observed and recorded by an oscilloscope (Tektronix Inc., MSO2024). The grounds of the electronic equipment were connected to the external electrode.

## Experimental procedure

**Figure 4:**
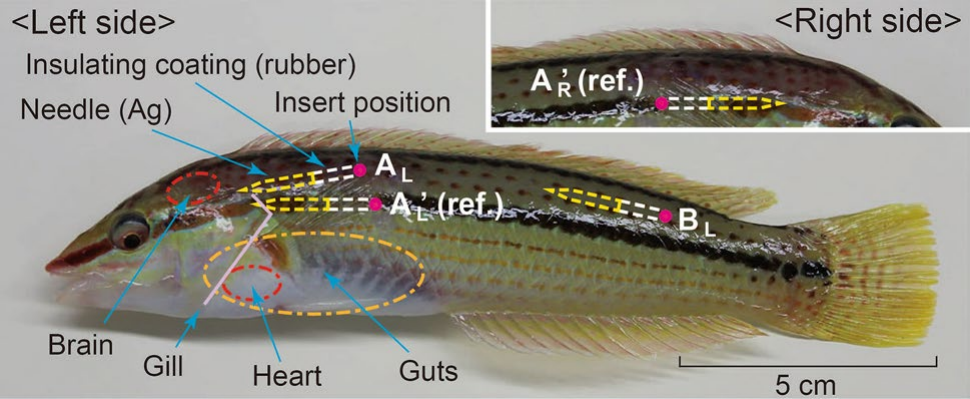
Fish (*Parajulis poecilepterus*) used as the experiment subject, and insert positions of the internal electrodes.

Three fish (*Parajulis poecilepterus*, total length of about 20 cm) were subjected to practical bioelectric measurements (Fig. 4). From the viewpoint of animal welfare, the minimum necessary number of subjects that allows the principle of the proposed method to be demonstrated was used for the measurements. This species is commonly distributed in shallow waters near the coast of Japan. To keep the subjects in easy (resting) state during the experiment, they were preliminarily kept in a tank containing 55 L of natural seawater at 20 ± 2 °C for two weeks before the experiment. Coral sand was spread over the aquarium bottom, and the natural seawater was purified by protein skimmer (Kamihata Fish Industries Ltd., Kaidou Daruma). During the keeping period, the fish were observed to spend most of the day sleeping in the sand and during the day time occasionally emerge from the sand and swim slowly.

Immediately before the experiment, one of the three fish was taken out of the seawater in the tank, and the internal electrodes were inserted into the fish’s muscle in the direction from tail to head; i.e., the signal line was wired toward the tail fin. This electrode configuration allows the fish to swim relatively freely and easily. To verify the availability of the proposed method, two insert positions of the internal electrodes were chosen: *A*_*L*_ (above the gills on the left side of the fish) and *B*_*L*_ (above the lateral line of the tail on the left side), as shown in Fig. 4. These positions are considered safe in terms of avoiding possible injuries to the brain, guts, and other tissues of the fish due to inserting the internal electrodes, and they are relatively near and far, respectively, from the cardiatic part of the fish. Moreover, to compare the proposed method with the conventional method, a pair of internal electrodes, namely, *A’*_*L*_ (below *A*_*L*_) and *A’*_*R*_ (opposite side of *A’*_*L*_), were also inserted into the fish. Incidentally, to measure bioelectric signals by two independent circuit systems, instead of inserting one common internal electrode et al., different internal electrodes were inserted et al. and *A’*_*L*_. This procedure for inserting the internal electrodes into the fish (removed from seawater) was completed in several minutes or shorter.

About ten minutes after the fish with the inserted internal electrodes was put into the tub, the fish stopped swimming and started resting (the body was stationary and only the gills were moving). In this resting state, resistance between the bioelectrodes was measured by using a specialized resistance meter. From the obtained resistances, the conditions of the inserted internal electrodes were checked. When the internal electrode was inserted appropriately, i.e., the conductor at the tip of the internal electrode was insulated from the seawater, the resistance between the internal electrode inserted into the fish and the external electrode was more than twice that between the internal electrode left in the seawater and the external electrode (Table 1).

**Table 1:** Typical resistances between bioelectrodes.

Bioelectrode	Resistance (kΩ) Subjects A, B, and C		
Internal electrode (*A*_*L*_) vs external electrode	1.1	1.0	1.2
Internal electrode (*B*_*L*_) vs external electrode	1.4	0.9	1.0
Internal electrode (*A'*_*L*_) vs internal electrode (*A'*_*R*_)	1.9	1.8	1.8
Internal electrode in seawater vs external electrode	0.4	0.3	0.3

After the above-described experimental preparations were completed, bioelectric signals of the fish while it was resting and moving were measured by the oscilloscope. To measure reactions of the alert fish, a visual stimulus was intentionally applied by moving a hand close to the fish’s eyes above the surface of the seawater, to make the resting fish temporarily swim.

All experiments in this study were performed in accordance with the Guidelines for Animal Experimentation of Osaka Prefecture University, Japan. Moreover, the laboratory fish were kept under the FELASA EUROGUIDE and treated based on the AVMA guideline (2013 Edition). Incidentally, according to Japanese law (Act on Welfare and Management of Animals), fish are not included in the list of experimental animals; thus, unfortunately there are no relevant institutional and national guidelines for the care and use of laboratory fish.

## Results and discussion

**Figure 5:**
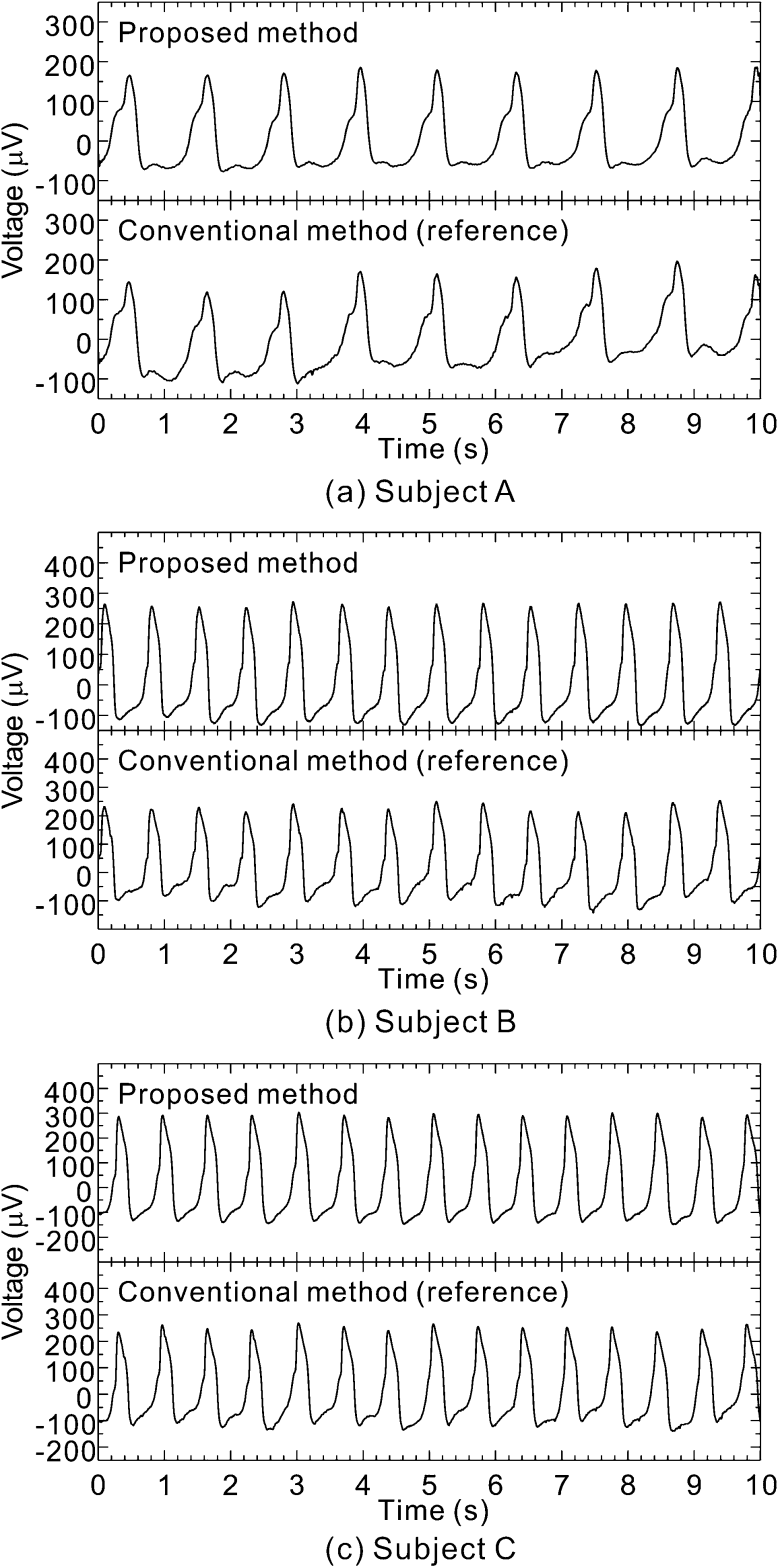
Examples of bioelectric signals acquired from above the gills when the three fish (subjects A, B, and C) were resting.

Examples of the bioelectric signals obtained from near the gills of three (fish) subjects (resting state) are shown in shown in Fig. 5a–c. In each figure, the upper and lower graphs represent signal waveforms detected by the proposed method (between internal electrode *A*_*L*_ and the external electrode) and the conventional method (between internal electrodes *A'*_*L*_ and *A'*_*R*_), respectively. Both signal waveforms were measured simultaneously and treated by a high-pass filter with cutoff frequency of 0.53 Hz and a low-pass filter with cutoff frequency of 30 Hz. The ordinate axes of these graphs represent voltage values before being amplified by the bioelectric amplifier. For all subjects, these signal waveforms continued during the experiments for over 1 h. Incidentally, the amplitudes of these signal waveforms do not change much even when the external electrode is placed at different positions in the tub. This phenomenon occurs because the resistance between the external and internal electrodes left in seawater is almost constant, i.e., 0.3 to 0.4 kΩ, regardless of the electrodes’ positions in the seawater in the tub (Table 1).

**Figure 6:**
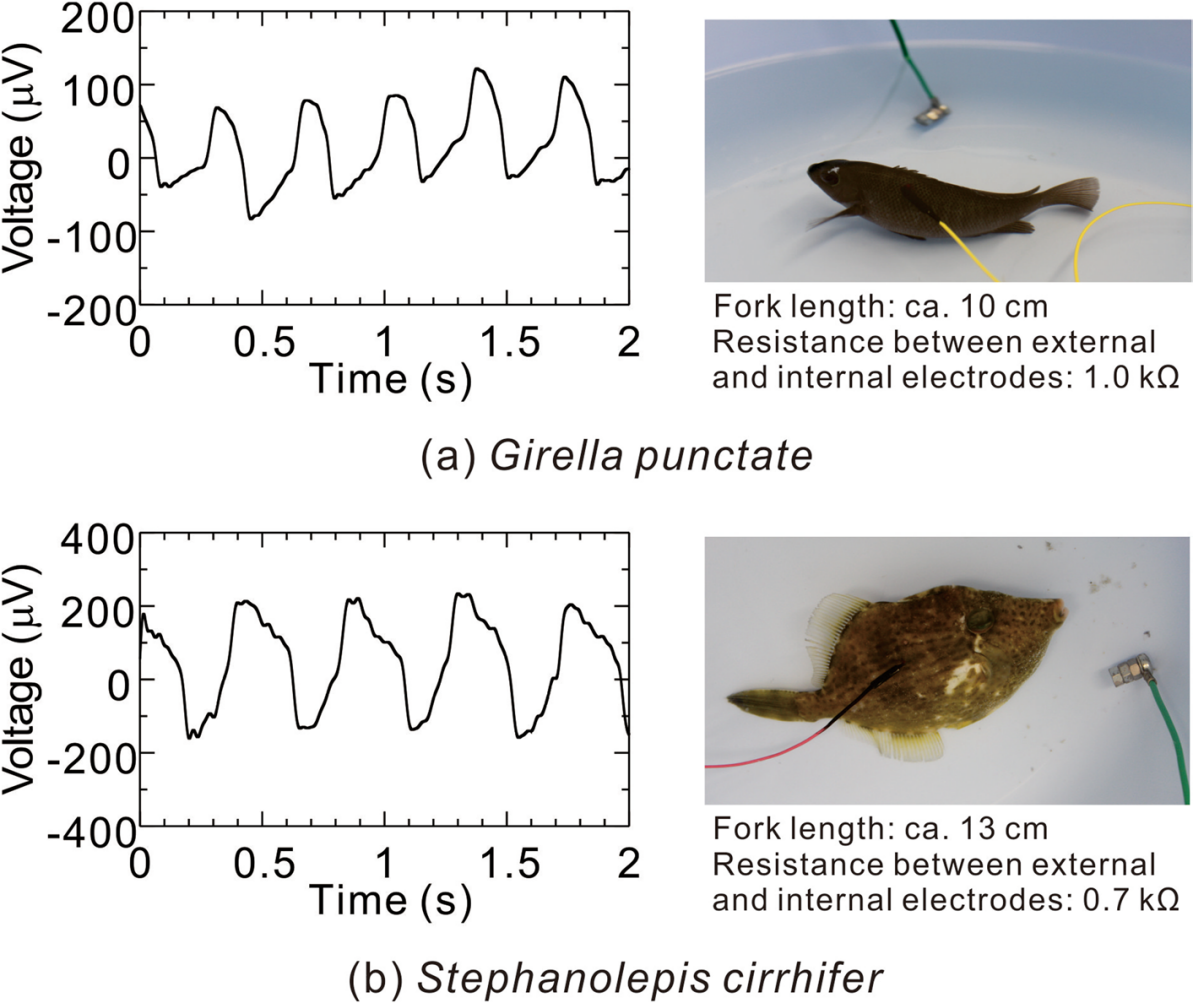
Examples of ECGs of other fish species and photographs of their subjects during the measurements.

As shown in Fig. 5a for subject A, the upper and lower waveforms closely correspond, and the waveform peaks are regularly arranged at intervals of about 1.2 s as 0.45, 1.65, 2.80, 3.95, 5.14 , … 9.93 s. As shown in Fig. 5b for subject B or 5c for subject C, the waveform peaks are also regularly arranged (although at different intervals to those of subject A). These regular periodicities of the waveforms indicate electrocardiograms (ECGs) as electric signals generated from living bodies. Therefore, by inserting one internal electrode above the fish’s gill, an ECG can be measured by the proposed method in a similar manner to the conventional method using two electrodes. More specifically, as shown in these figures, for all the subjects (fish), the amplitude of the signal waveform obtained by the proposed method is larger than that obtained by the conventional method. This result shows that depending on the insertion position of the internal electrode, the proposed method has higher signal sensitivity than the conventional method. Incidentally, it was also confirmed that the ECGs of other fish species could be measured by the proposed method (Fig. 6).

**Figure 7:**
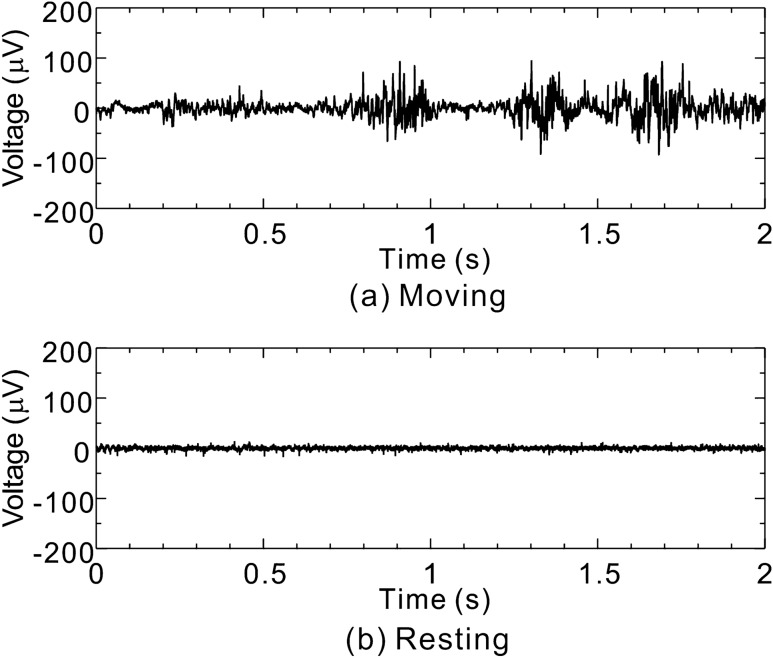
Examples of bioelectric signals measured by the proposed method at the fish’s tail.

Examples of bioelectric signals detected by the proposed method at the fish’s tail are shown in Fig. 7a and b. These figures represent bioelectric signals of the fish while it was moving and resting, respectively. These signal waveforms were treated by a high-pass filter with cutoff frequency of 10 Hz, a low-pass filter with cutoff frequency of 1000 Hz, and a notch filter with cutoff frequency of 60 Hz (commercial frequency). Note that because the low-pass and notch-filtering treatment could not be applied by the bioelectric amplifier itself, on the basis of bioelectric signals recorded by the oscilloscope, digital signal processing was also performed on a personal computer.

As shown in Fig. 7a, when the fish was moving, rapidly changing signal waveforms were observed at around 0.9, 1.3, and 1.7 s. Similar fluctuating signal waveforms were observed in the case of the other two fish as well. It was visually confirmed that when the rapidly changing signal waveform appeared, the fish’s tail was moving. On the contrary, as shown in Fig. 7b, when the fish was in resting state, a rapidly changing waveform as shown in Fig. 7a was not observed; however, a uniform waveform with voltage width of ± 10 µV_p–p_ was observed. Of course, it was confirmed that while such a uniform voltage was observed, the fish didn’t move its tail. It is concluded from this result that such rapidly changing waveforms must be electromyograms (EMGs).

The three fish survived in the aquarium for two weeks or longer after the bioelectric experiments were completed, even though the internal electrodes inserted into their living body were relatively large size. This finding suggests that physical stress of marine animals can be lowered by developing and fabricating a very small internal bioelectrode, which could then be used with the proposed method for monitoring the health of farmed fish.

## Conclusion and future work

Through experiments in which a fish species (*Parajulis poecilepterus*) was chosen as a marine animal subject, we established a novel method of bioelectric (ECG and EMG) measurements utilizing the conductivity of seawater. This method is very simple because only one needle-type electrode is inserted into the marine animal under study at each measurement point.

To implement the novel method practically, the exact location of the bioelectrode inserted in the living body will be determined by using X-ray measurement, and then bioelectric signals of various kinds of marine animals will be measured in a real marine environment.

## Data Availability

The data that support the findings of this study are available from the corresponding author upon reasonable request.
